# 643. Preoperative Burn Wound Antisepsis: A Survey Study of Burn Surgeon Practices

**DOI:** 10.1093/jbcr/irag033.480

**Published:** 2026-04-06

**Authors:** Megan E Daniels, Stephanie E Shin, Lud H Eyasu, Franklin R Gergoudis, Rendell Bernabe, Monica Jinsi, Zachary Brown, Prabhu Senthil-Kumar, Michael J Feldman

**Affiliations:** Evans-Haynes Burn Center at the VCU Medical Center/Virginia Commonwealth University, Richmond, VA; Evans-Haynes Burn Center at the VCU Medical Center/Virginia Commonwealth University, Richmond, VA; Evans-Haynes Burn Center at the VCU Medical Center/Virginia Commonwealth University, Richmond, VA; Evans-Haynes Burn Center at the VCU Medical Center/Virginia Commonwealth University, Richmond, VA; Evans-Haynes Burn Center at the VCU Medical Center/Virginia Commonwealth University, Richmond, VA; Evans-Haynes Burn Center at the VCU Medical Center/Virginia Commonwealth University, Richmond, VA; Evans-Haynes Burn Center at the VCU Medical Center/Virginia Commonwealth University, Richmond, VA; Evans-Haynes Burn Center at the VCU Medical Center/Virginia Commonwealth University, Richmond, VA; Virginia Commonwealth University, Richmond, VA

## Abstract

**Introduction:**

Antiseptic preparation of surgical sites is a standard practice to decrease microbial load and prevent infection. However, in burn surgery, open wounds pose unique challenges, and there is no clinical standard for the use of chlorhexidine or iodine-based solutions. This study aims to assess current practices and preferences in preoperative antisepsis among burn surgeons.

**Methods:**

At the authors' request, the American Burn Association (ABA) distributed a survey to attending burn surgeons in April 2025. The survey focused on standard practices and hospital protocols for preoperative burn wound antisepsis before the initial surgical excision after injury.

**Results:**

A total of 43 surgeons (43/499; 9%) from 36 different burn centers participated, representing 19 U.S. states and two Canadian provinces (see Fig. 1). Most centers (n = 27; 75%) have current ABA verification. The largest subgroup consisted of attending surgeons with over 20 years of experience (n = 16; 37%), followed by those with less than 5 years (n = 12; 28%). Fewer than half (n = 19; 44%) reported having institutional guidelines for surgical antisepsis, which included considerations like anatomic location (n = 14; 74%), burn depth (n = 3; 16%), total body surface area (n = 2; 11%), and the type of planned reconstruction, such as autologous skin grafts or other allograft coverage (n = 5; 26%). The most cited guideline was to avoid chlorhexidine-based products on the face, neck, and scalp (n = 12; 63%). Among respondents without institutional guidelines (n = 24; 56%), the most cited factor influencing antiseptic choice was anatomical location (n = 15; 63%), with many surgeons avoiding chlorhexidine-based products on the face, neck, and scalp. Secondary considerations included cost and availability (n = 5; 21%) and the type of burn reconstruction planned (n = 5; 21%).

**Conclusions:**

There is considerable variation in presurgical burn wound antisepsis among attending surgeons, although anatomic site may significantly influence institutional guidelines and surgeon preferences.

**Applicability of Research to Practice:**

This study highlights the variability in preoperative burn wound antisepsis. Further research into surgeon practices and their impact on surgical outcomes is necessary to identify if one agent is superior, helping to guide the best wound bed antisepsis before surgical excision.

**Funding for the study:**

N/A.

